# A Clinical Audit of Operation Notes Documentation and the Impact of Introducing an Improved Proforma: An Audit Cycle

**DOI:** 10.7759/cureus.50281

**Published:** 2023-12-10

**Authors:** Rao Erbaz Hassan, Ismail Akbar, Arif Ullah Khan, Muhammad Bilal Hameed, Muhammad Raza, Syed H Shah, Syed H Shah, Hamza Haroon, Rao Aizaz Hanzala

**Affiliations:** 1 Department of Surgery, Ayub Teaching Hospital, Abbottabad, PAK; 2 Department of Surgery, Khyber Teaching Hospital, Peshawar, PAK; 3 Department of General Surgery, Ayub Medical College, Abbottabad, PAK; 4 Department of Emergency Medicine, Russells Hall Hospital, Dudley, GBR; 5 Department of Orthopaedics and Trauma, Pakistan Institute of Medical Sciences, Islamabad, PAK; 6 Department of Surgery, Khyber Medical College, Peshawar, PAK

**Keywords:** quality improvement projects, proforma, audit, operation notes, surgery

## Abstract

Introduction

Accurate and comprehensive documentation of surgical procedures is vital in healthcare for both medical and legal purposes. This audit assessed adherence to international guidelines for operative note documentation in a general surgery department and the impact of introducing educational initiatives and an enhanced proforma.

Methods

A retrospective audit of 100 operative notes was conducted in April 2023, followed by a prospective re-audit of another 100 notes in October-November 2023. A checklist based on Royal College of Surgeons (RCS) guidelines assessed 20 parameters. An improved proforma and an awareness session for surgeons were implemented between audits. Data analysis utilized the IBM SPSS Statistics for Windows, Version 26.0 (Released 2019; IBM Corp., Armonk, New York, United States). A paired-sample t-test was used, and a p-value < 0.001 was considered statistically significant.

Results

The initial audit revealed discrepancies in documentation, with missing information on deep vein thrombosis (DVT) prophylaxis, elective/emergency settings, anticipated blood loss, closure technique specifics, and prosthesis/mesh details. Legibility was satisfactory in 88% of notes. After implementing the proforma and awareness session, significant improvements were observed in all parameters, with documentation rates exceeding 91%. Overall documentation completeness increased from 65.2% to 95.2%. Results of the paired-sample t-test indicated a significant difference before and after the introduction of the new proforma (Mean (M) = 65.2, standard deviation (SD) = 34.3 versus M = 95.2, SD = 4.3) with a p-value of 0.0005.

Conclusion

Regular audits, surgeon education, and standardized proformas are essential for maintaining high standards in operative note documentation, contributing to improved patient care and safety.

## Introduction

Accurate documentation of patient management is essential for healthcare providers, serving the dual purposes of facilitating scientific evaluations and providing a legal defense against allegations of medical negligence. Medical records play a pivotal role in analyzing treatment outcomes, devising protocols, and substantiating insurance claims [1]. Thorough documentation of operative notes holds immense significance, carrying both medical and legal implications. The implementation of educational initiatives for surgeons, coupled with the provision of documentation aids in the form of proforma, has demonstrated significant enhancement in adhering to the guidelines set by the Royal College of Surgeons of England (RCS) [2].

Domain 1.3 of the Good Surgical Practice guidelines, formulated by the RCS, emphasizes the proper documentation of any procedure performed and outlines specific parameters that must be documented for every operative procedure [3]. Operative notes provide healthcare professionals with essential insights into procedural details and postoperative care requirements. Surgeons make critical decisions regarding thromboprophylaxis and antibiotic administration based on individual cases, underscoring the pivotal role of operative note quality in optimizing surgical patient management [4].

This audit, conducted in a tertiary care hospital, aimed to evaluate adherence to international guidelines for operation notes documentation in the surgical department. The study also assessed the impact of educational initiatives for surgeons, alongside the implementation of a new proforma designed to provide documentation aid to the surgeon.

## Materials and methods

This cross-sectional study was conducted in the General Surgery Department of a tertiary care hospital, Ayub Teaching Hospital, Abbottabad, Pakistan. It was approved by the Medical Teaching Institute Abbottabad Institutional Ethical Review Committee (Approval number: RC-EA-2023/060). The objective was to assess operative note quality at a tertiary care hospital against RCS standards. The aim was to identify missing details, incompleteness, and deviations to determine the necessity for adjustments to the proforma format.

One hundred consecutive operation notes were audited in both cycles. The central focus was improving documentation rates for specific parameters. Data collection for the first cycle (April 1-30, 2023) was retrospective and that for second cycle (October 1-November 30, 2023) was prospective. A checklist was used to evaluate 20 parameters in line with Good Surgical Practice guidelines from the RCS [3]. All General Surgery patients were included without exclusion criteria based on procedure type. A sole reviewer meticulously examined all operation notes, gathering feedback on legibility from nursing staff and doctors.

The collected data underwent analysis and presentation to the local audit committee. The findings led to the implementation of an enhanced proforma, introducing a new version. An awareness session targeted individuals (residents and consultant surgeons) actively involved in the notes documentation process. The new proforma's use became mandatory, discontinuing the old version. A second dataset was collected, and results were presented to the local audit committee, expressing satisfaction with the improvements, and completing the audit cycle.

Data analysis used the IBM SPSS Statistics for Windows, Version 26.0 (Released 2019; IBM Corp., Armonk, New York, United States). A paired-sample t-test was used and a p-value < 0.001 was considered statistically significant. All 20 parameters served as variables in this study.

## Results

In the initial audit conducted in April 2023 (Table 1), 100 operative notes were retrospectively analyzed. Patient identification, surgeon and assistant names, operative procedure, operative diagnosis, operative findings, and antibiotic prophylaxis were documented in over 90% (n= 90) of the notes, establishing them as the most frequently recorded parameters. Conversely, deep vein thrombosis (DVT) prophylaxis and details regarding elective/emergency settings were absent in all 100 notes. Parameters such as anticipated blood loss, closure technique specifics, and prosthesis/mesh details were documented in less than 40% (n= 40) of operative notes, marking them as the least documented aspects. A legibility assessment revealed that approximately 88% (n= 88) of the notes were satisfactory, while 12% (n= 12) were considered poorly legible. In summary, these operative notes documented 65.2% (standard deviation (SD) = 34.3) of all parameters.

**Table 1:** Comparison between documentation frequency of parameters in initial audit and re-audit The data has been represented as percentage (%); n: Total number of notes applicable for a specific parameter P value < 0.001 DVT: deep vein thrombosis

Parameter Assessed	Documentation frequency in initial audit cycle (n)	Documentation frequency in the re-audit cycle (n)	Change	P-value
Identification	96% (100)	97% (100)	1%	0.3
Time of procedure	62% (100)	95% (100)	33%	<0.001
Elective/emergency procedure	0% (100)	84% (100)	84%	<0.001
Name of surgeon and assistant	98% (100)	100% (100)	2%	0.15
Name of theatre anaesthetist	89% (91)	95% (100)	6%	0.013
Name of procedure	91% (100)	100% (100)	9%	0.002
Type of incision	79.38% (97)	91% (100)	11.62%	<0.001
Operative diagnosis	93% (100)	98% (100)	5%	0.08
Operative findings	90% (100)	99% (100)	9%	<0.001
Complications encountered	76.34% (93)	97% (100)	20.66%	<0.001
Any extra procedure performed with reason	80.4% (92)	96% (100)	15.6%	<0.001
Details of tissue removed, altered or added	75% (100)	94% (100)	19%	<0.001
Details of closure technique	38% (100)	96% (100)	58%	<0.001
Anticipated blood loss	2% (100)	97% (100)	95%	<0.001
Prosthesis/mesh details	18% (11)	85% (20)	67%	<0.001
Antibiotic prophylaxis	95% (100)	94% (100)	-1%	0.3
DVT prophylaxis	0% (19)	94% (100)	94%	<0.001
Detailed post op instructions	76% (100)	96% (100)	20%	<0.001
Signature	57% (100)	99% (100)	42%	<0.001
Legibility	88% (100)	97% (100)	9%	0.002

An evaluation of 100 operative notes was conducted in the re-audit from October to November 2023 (Table 1). This re-audit revealed significant improvements in various parameters. The procedure date and the names of the surgeon and assistant were documented in all notes, while elective/emergency procedures and prosthesis/mesh details were noted in 84% (n= 84) and 85% (n= 85) of the notes, respectively. All other parameters were documented in over 91% (n= 91) of the notes, marking substantial success. In summary, 95.2% (SD = 4.3) of all parameters were documented in the re-audit, reflecting a cumulative increase of 30% from the initial audit.

Results of the paired-sample t-test indicated that there is a significant large difference in documentation quality of operative notes before and after the introduction of the new proforma (Mean (M) = 65.2, SD = 34.3 versus M = 95.2, SD = 4.3) with a P-value of 0.0005.

## Discussion

Ensuring precise patient record-keeping is imperative in the medical field, especially with the escalating frequency of complaints related to medical malpractice and negligence. Operative notes hold a pivotal position in patient care, education, and research, establishing them as a crucial component [5]. This audit thoroughly investigated one of the most essential documents in the overall landscape of medical practice, highlighting its specific significance in the realm of surgical care.

In our hospital, a unified, standardized operation notes proforma is utilized across all surgical departments. In this audit, it seemed feasible to modify this proforma according to guidelines to enhance the documentation quality in operation notes. Khalid et al. employed a similar strategy in their audit of surgical operation notes, covering the same surgical procedures. The implementation of an enhanced proforma format significantly improved overall documentation accuracy, increasing details recorded from 58.6% to 95.3% in the notes [6]. The findings of this audit, coupled with the evidence presented, highlight the substantial enhancement achievable in patient care through the implementation of straightforward measures. These include targeted education on operative notes documentation and the introduction of an enhanced proforma. A comparable successful strategy was observed in a study by Ahmed et al., where all components of chest drain insertion notes demonstrated significant improvement in the subsequent audit [7].

**Figure 1:**
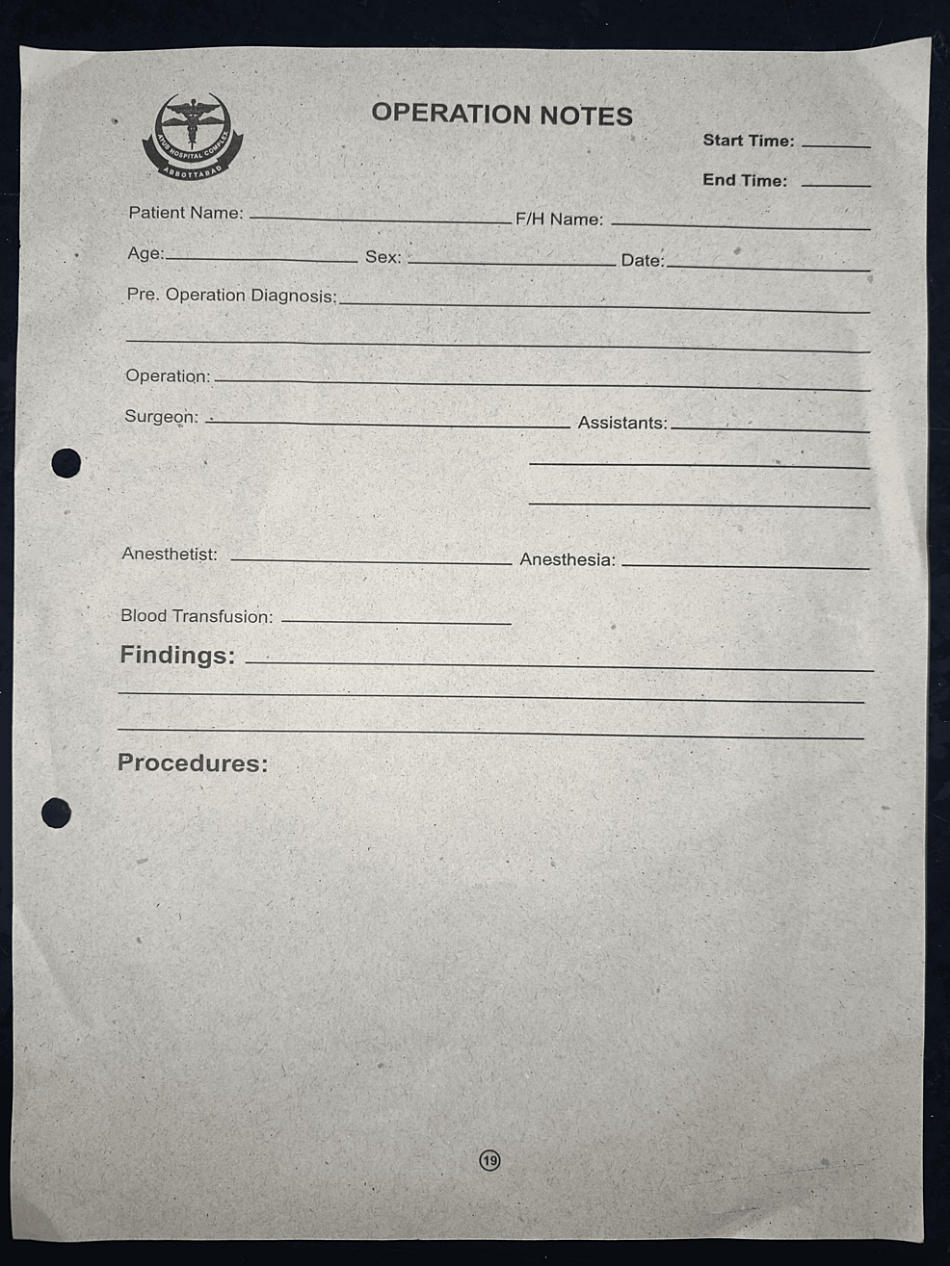
Operative notes proforma previously used showing multiple shortcomings

**Figure 2:**
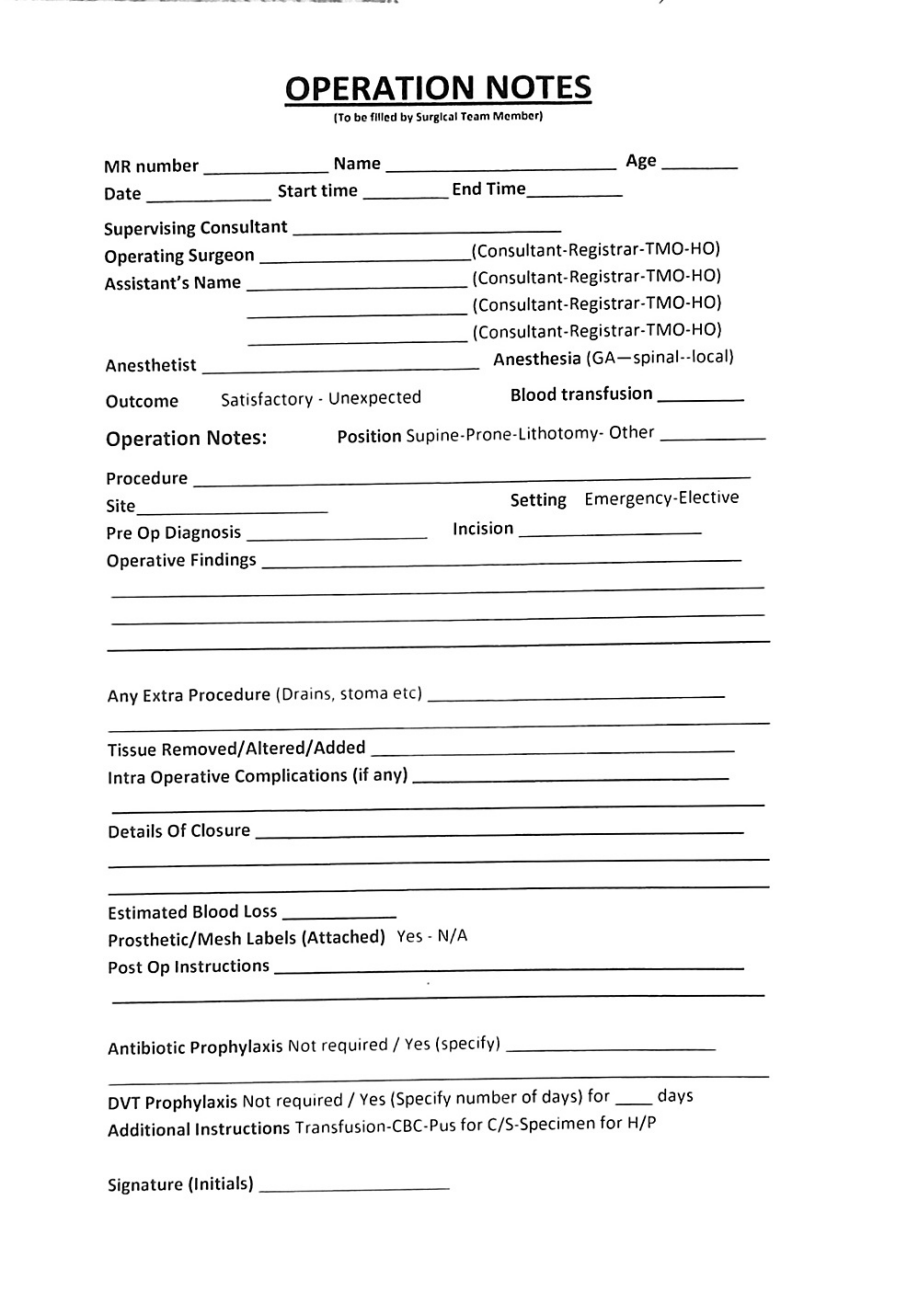
Revised operative notes proforma based on RCS guidelines RCS: Royal College of Surgeons

The initial audit of operative notes exposed various shortcomings in the documentation process. These involved deficiencies in the proforma that was being used for documentation and a lack of knowledge regarding the guidelines for proper documentation (Figure 1). After this, the findings were presented to the local audit committee, resulting in several recommendations. The operative notes proforma underwent a redesign, introducing a section for additional parameters not included in the earlier version (Figure 2). These additional parameters encompassed the classification of emergency/elective procedures, type of incision, details of tissue alterations or additions, any extra procedures performed with reasons, antibiotic and DVT prophylaxis, complications, anticipated blood loss, closure technique specifics, prosthesis/mesh details, and signature. This modification has previously demonstrated enhanced efficiency in documentation [8].

Utilizing a clear and comprehensive note is crucial to prevent avoidable delays or errors in postoperative management [9]. However, assessing whether enhancing the quality of operative notes genuinely translates to improved patient outcomes poses a significant challenge. This difficulty is commonly encountered in studies aiming to elevate the quality of operative notes. Additional research is necessary to ascertain the actual impact of high-quality surgical notes on patient outcomes.

The critical challenge of legibility persists in handwritten notes. Research consistently supports the effectiveness of computer templates or typed notes over their handwritten counterparts [10]. Due to financial constraints, our hospital has yet to adopt electronic operation notes. Nevertheless, there is a firm conviction that electronic note documentation represents the pinnacle of legibility standards.

The audit’s limitations include a focus on a single hospital, potentially limiting generalizability. The study primarily evaluates the impact of a new proforma but may not consider concurrent changes in clinical practices and patient outcomes. Emphasis on quantitative parameters might overlook qualitative aspects of documentation quality.

This audit sheds light on opportunities to enhance surgeons' documentation practices in the future. In the course of this audit cycle, it was observed that assessing practices against established standards and subsequent re-auditing resulted in a substantial improvement, aligning with findings reported by other researchers [7-9].

## Conclusions

The study demonstrates a significant enhancement in operative note quality by implementing recommendations from an initial audit, underscoring the importance of regular audits in maintaining documentation standards. Improving precision in operating room documentation through surgeon education and proforma use, aligned with RCS guidelines, is effective. Continuous audits, especially among trainees, are vital for refining practices, ensuring patient safety, and upholding the highest quality of care.

## Figures and Tables

**Figure 3:**
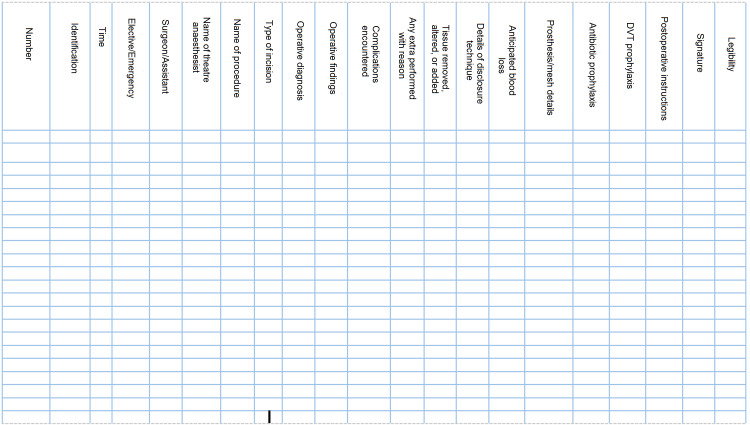
Checklist used for the evaluation of 20 parameters
